# Impaired Oculomotor Behavior of Children with Developmental Dyslexia in Antisaccades and Predictive Saccades Tasks

**DOI:** 10.3389/fpsyg.2016.00987

**Published:** 2016-06-30

**Authors:** Katerina Lukasova, Isadora P. Silva, Elizeu C. Macedo

**Affiliations:** ^1^Faculty of Psychology, University Cruzeiro do SulSao Paulo, Brazil; ^2^Neuroimagem Funcional, LIM-44, Faculty of Medicine, University of Sao PauloSao Paulo, Brazil; ^3^Social and Cognitive Neuroscience Laboratory and Developmental Disorders Program, Center for Health and Biological Sciences, Mackenzie Presbyterian UniversitySao Paulo, Brazil

**Keywords:** dyslexia, eye movements, implicit learning, antisaccades, predictive saccades

## Abstract

Analysis of eye movement patterns during tracking tasks represents a potential way to identify differences in the cognitive processing and motor mechanisms underlying reading in dyslexic children before the occurrence of school failure. The current study aimed to evaluate the pattern of eye movements in antisaccades, predictive saccades and visually guided saccades in typical readers and readers with developmental dyslexia. The study included 30 children (age *M* = 11; *SD* = 1.67), 15 diagnosed with developmental dyslexia (DG) and 15 regular readers (CG), matched by age, gender and school grade. Cognitive assessment was performed prior to the eye-tracking task during which both eyes were registered using the Tobii® 1750 eye-tracking device. The results demonstrated a lower correct antisaccades rate in dyslexic children compared to the controls (*p* < 0.001, *DG* = 25%, *CC* = 37%). Dyslexic children also made fewer saccades in predictive latency (*p* < 0.001, *DG* = 34%, *CG* = 46%, predictive latency within −300–120 ms with target as 0 point). No between-group difference was found for visually guided saccades. In this task, both groups showed shorter latency for right-side targets. The results indicated altered oculomotor behavior in dyslexic children, which has been reported in previous studies. We extend these findings by demonstrating impaired implicit learning of target's time/position patterns in dyslexic children.

## Introduction

Dyslexia is defined according to the International Dyslexia Association (IDA) as a “specific learning disability that is neurological in its origin. It is characterized by difficulties with accurate and/or fluent word recognition, and by poor spelling and decoding abilities.” These difficulties are believed to stem from a deficit in the phonological component of language that is often unexpected in relation to other cognitive abilities and the provision of effective classroom instruction. Secondary consequences may include problems in reading comprehension and reduced reading experience that can impede the development of vocabulary and background knowledge (Lyon et al., 2003).

In addition to impairment in phonological processing, some studies have noted other rather subtle deficits in motor and perceptual domains, such as manual finger tasks (Birkett and Talcott, 2012), balance (Stoodley and Schmahmann, 2009), low-frequency perception (Bednarek et al., 2006), visual vergence (Bucci et al., 2008a) and eye movements during reading and motor tasks (Jones et al., 2008; Kronbichler et al., 2009).

The analysis of movements during the reading of typical readers indicates that the eyes make short saccades from left to right, alternating with fixations and occasional regression from right to left (Olitsky and Nelson, 2003; Yokomizo and Lukasova, 2008; Oliveira et al., 2013). The pattern of eye movements found in dyslexic compared to normal readers presented a greater number of saccades, longer fixation periods and a greater number of regressions (Ogusuko et al., 2008; Bellocchi et al., 2013). So far, it has been inconclusive whether the altered oculomotor pattern is due to a more general oculomotor deficit or is byproduct of the struggle with written text. The later is supported by a study finding impaired eye-tracking patterns both in dyslexic and normal readers when reading a text significantly above their reading skills (Rayner, 1998). On the other side, some studies reported altered eye-movement patterns in dyslexics while preforming oculomotor tasks without written content (De Luca et al., 1999). Thus, a better understanding of the oculomotor behavior in different eye-tracking tasks can enable greater comprehension of underlying cognitive functions, such as perception, attention, executive functions, procedural learning and others.

The advantage of using eye-tracking tasks in developmental impairments such as dyslexia is the analysis of saccadic properties. In unimpaired children, saccadic speed or adaptation are on the same level as adults by the early childhood, whereas others, such as latency, precision and gain, mature in early adulthood (Klein et al., 2005; Eenshuistra et al., 2007; Seassau and Bucci, 2013). According to some studies, this developmental course reflects the gradual cortical maturation and reorganization of neural communication in regions involved in oculomotor control (Velanova et al., 2008).

Oculomotor behavior in normally developing children has been assessed for the basic properties of the oculomotor system, such as vergence, visual adaptation, visually guided saccades (Biscaldi et al., 2000), and others aiming at more complex components of cognition, such as antisaccades (Velanova et al., 2009), predictive saccades (Ross and Ross, 1987; Liddle et al., 2009), smooth pursuit (Gardner and Lisberger, 2001), and memory-guided saccades (Geier et al., 2009). Over the past decades, inconsistent findings were reported on the oculomotor deficit in children with developmental dyslexia. Altered performance of dyslexics was found in: number of error and correction rate in antisaccades (Biscaldi et al., 2000), vergence (Bucci et al., 2009), binocular coordination (Bucci et al., 2008b), and pursuit (Eden et al., 1994). These deficits were mostly linked to impaired visual attention and/or altered motor learning mechanisms (Bucci et al., 2008a,b; Goswami, 2014).

The procedural motor learning can be studied with square-wave tracking of a visual stimulus alternating at a constant pace between fixed left and right positions. A subject who is instructed to follow the target with his eyes implicitly perceives this stimulus time and position regularity and quickly reduces his saccadic latency to zero or to negative values generating so called predictive saccades (Shelhamer and Joiner, 2003; Isotalo et al., 2005; McDowell et al., 2008). Thus, predictive saccades represent a quick shift from visually guided to internally guided behavior. The generation of predictive saccades is thought to be based on the previous trials timing representation, the memory of the previous saccade intervals and error analyses of the past and future eye movements (Joiner and Shelhamer, 2006). In short, these are the same mechanisms of error detection and processing that drive motor adaptation which has been increasingly important for studying motor learning in general (Wong and Shelhamer, 2011).

The motor adaptation is commonly studied by task called *intrasaccadic step*, during which the saccade targert is repeatly displaced in one direction and after a few trials the subject naturaly compensate for the object's displacement. However, this kind of tasks are generally long, monotonous and fatiguing being thus little suitable for clincal popullation and developmental studies. Therefore, a distinct advantage of a square-wave tracking over intrasaccadic step task is its duration and additionally a fact that the average saccadic amplitude remains constant, differently from changing movement gain in response to the targets errors (Wong and Shelhamer, 2011).

A major motivation for this study was to test a motor learning model through eye movements measured during square-wave task in children with developmental dyslexia and controls. Besides that, we evaluated saccadic movements through visually guided saccades and antisaccades. Visually guided saccade, also known as a pro-saccade, is a simple eye movement to the stimulus in order to align the fovea with the target, while antisaccades involves inhibition of this prepotent response and generation of a saccadic movement to the opposite direction of the peripheral cue (McDowell et al., 2008). The aim of this study was to compare the patterns of eye movements in internally guided saccades (predictive saccades), visually guided saccades and volitional saccades with inhibition (antisaccades).

Based on the available studies with dyslexic population described above, we expected to find between group differences in internally (volitionally) guided saccades (predictive and antisaccades) but not in visually guided saccades (Eden et al., 1994; Biscaldi et al., 2000).

## Materials and methods

### Subjects

The subjects of this study were 30 children between 8 and 13 years of age from different basic schools, ranging from 3rd to 8th grade (*M* = 6th grade; *SD* = 1.64). The participants with dyslexia were recruited through the Brazilian Association of Dyslexia (ABD; http://www.dislexia.org.br) associated to International Dyslexia Association (IDA). The subjects had diagnosis confirmed by ABD and for the purpose of this study, an additional cognitive assessments was performed on all the subjects (reported in details in Toledo et al., 2014). Two scores were used as exclusion criteria: scoring below the 25th percentile on the Wechsler Intelligence Scale for Children III (WISC-III, Wechsler and Figueredo, 2002) and on sustained attention test.

### Instruments

**Figure 1:**
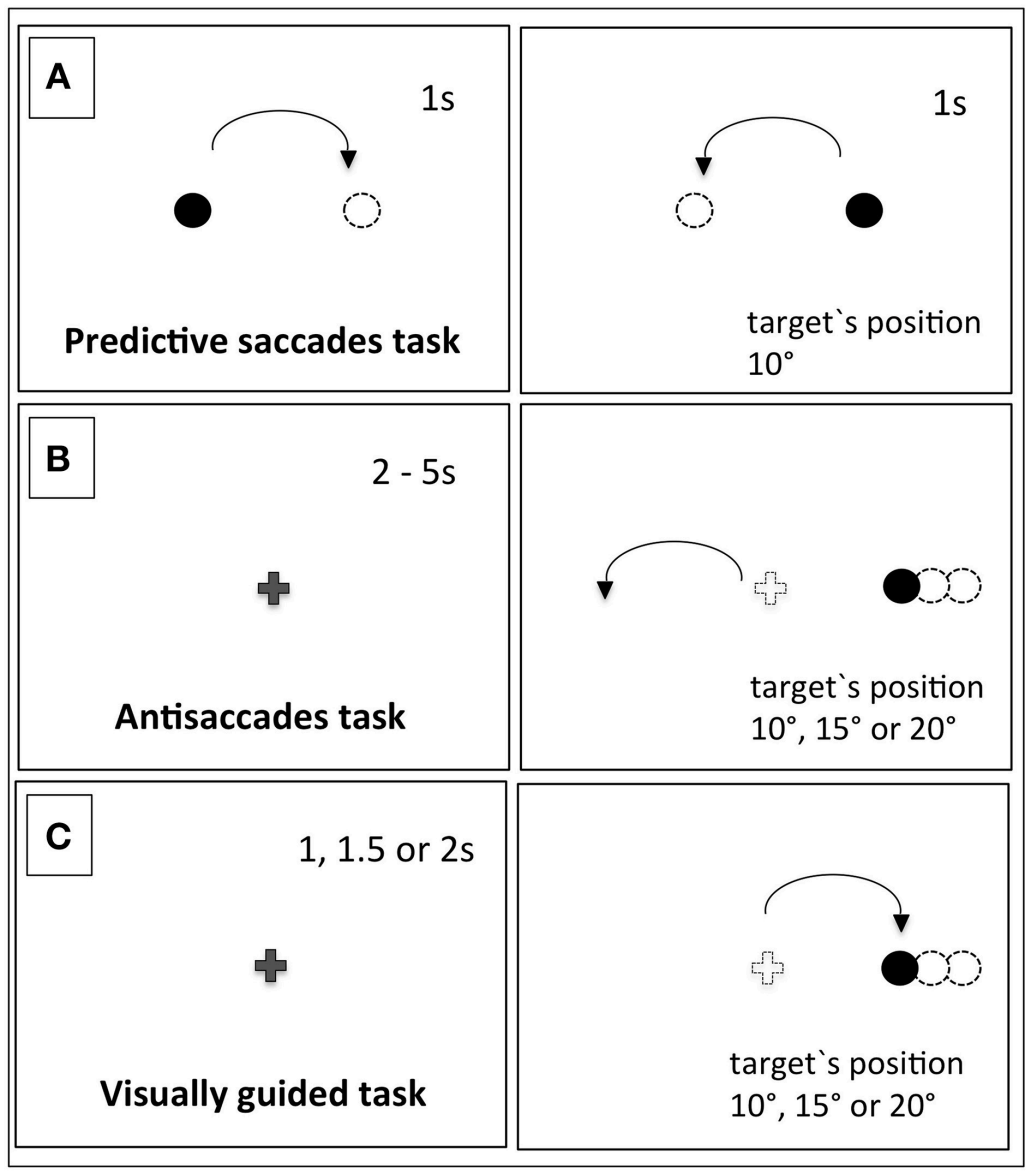
**The schematic picture of the task's design**. In task **(A)**, the predictive saccades task had constant time and stimuli positions. In task **(B)**, the antisaccades task required an eye movement in the opposite direction of the stimuli, which could appear in one of 3 different positions. In task **(C)**, the visually guided task saccades were triggered in the direction of the target, which could appear in one of 3 different positions.

Three oculomotor tasks with in-line eye tracking were administered to the subjects of this study at the Social and Cognitive Neuroscience Laboratory at Universidade Presbiteriana Mackenzie. The tasks are depicted in Figure 1.

#### Predictive saccades task (PS)

This task consisted of a series of 28 screens, each containing a point target either on the right or on the left. The subject had to visually track the stimuli presented in a square wave manner 10° to the right or left from the center with fixed duration of 1 s (Goldberg et al., 2002.). The point was black on the white background and had a diameter of 5 mm.

#### Antisaccades task (AS)

This task consisted of 30 screens initiated by the appearance of the central fixation cross during a random period of time that varied between 2 and 5 s. The peripheral target appeared to the right or left, in a position ranging from 10, 15, or 20 degrees from the center. The target's onset overlapped with the fixation cross offset. The subjects were instructed not to look at the peripheral target, but instead to perform saccades in the opposite direction (adapted from Luna and Sweeney, 2001; Goldberg et al., 2002). The central cross was 10 mm in length, and the peripheral point was 5 mm in diameter—both were black.

#### Visually guided saccades task (VG)

This task consisted of 45 screens initiated by the appearance of the central fixation cross and with its offset; the peripheral target appeared to the right or left after pseudo-randomized time intervals of 1, 1.5, or 2 s. The participants had make a saccade to the target's position, which could range from 10, 15, or 20 degrees on either the right or left side.

### Equipment

We used the computerized eye tracker Tobii® 1750 (Tobii Technology) for registering binocular eye movements. The equipment consists of a screen that is 17″ TFT 1280 × 1024 pixels, with two high resolution cameras having a wide field capture embedded in the bottom of the screen. The registration of binocular movements allows for tolerance of head movement (30 × 15 × 20 cm), without losing calibration or precision. If only one eye is in the field of capture, the equipment captures the motion of the eye and compensates for the temporary absence of the other eye.

### Procedures

This project was approved by the Ethics Committee in Research of the Universidade Presbiteriana Mackenzie. The data acquisition took place in a suitable environment for research, at the Social and Cognitive Neuroscience Laboratory. All participants were volunteers, and before enrolling in the study, their parents signed written informed consent approved by the Ethics Committee. Overall, the assessment was performed in two meetings of 90 min.

### Analysis

The data were analyzed with group as the between-subjects factor and task as the within-subjects factor. The cognitive data and saccadic latency from the eye movement tasks (PS, AS, and VG) were compared for significance with ANOVA.

In the PS task, the saccades were classified according to their saccadic latency, which is the time between the target on-set and the saccade generation. All saccades that happened with latencies between −300 and 300 ms were considered correct saccades. Saccades that were generated within 120–300 ms after the stimulus on-set were classified as regular. Saccades generated within 0–120 ms after the stimulus disappearance or within 300 ms before the stimulus disappearance were considered predictive (−300–120 ms). However, some studies set lower time limit for predictive saccades around 75–100 ms (Isotalo et al., 2005; Joiner and Shelhamer, 2006), we considered more liberal time limit of 120 ms due to the children population (McDowell et al., 2008). All the other saccades were considered errors. If an error saccades happened with latency < −300 ms they were classified as erro-antecipated, and if the latency was >300 ms, the saccades were considered error-late.

In the AS task, in addition to latency, we compared the rate of correct antisaccades, error saccades directed toward the target and corrected saccades that were pro-saccade followed by corrective antisaccade. Generalized linear mixed models with participant as a random effect were used to identify the association between the occurrence of each type of saccade and the group fixed effects. The models were estimated using the binomial link function and an adaptive Gauss-Hermite quadrature approximation to the likelihood function (Bates et al., 2015).

In order to visualize learning curve in PS task, the mean saccadic latencies within predictive and regular type were averaged for every target along the task and plotted for each group. To smooth the data, we used Cubic smooth spline in R (Green and Silverman, 1994) and estimated the confidence interval using 10,000 booth strap replicates.

Since one of the aims of this study was to see wheatear there was a between group difference in prediction learning, we estimate a conditional probability of remaining within predictive state. Predictive learning is ability to perceive the position and time regularity of the moving stimulus and reduce the saccadic reaction time to the predictive latency of below 80 ms (Fischer et al., 1997; Bucci and Seassau, 2014). Once the predictive behavior is established the tracking becomes independent and persist for some time even without the pacing stimulus (Joiner and Shelhamer, 2006). The efficiency of remaining within predictive behavior was estimated by conditional probability formula used by Joiner and Shelhamer (2006) where the probability of saccade *i* + 1 being predictive (S_*i* + 1_ = P) if saccades *i* is predictive (S_*i*_ = P) is:

$$\begin{array}{l} P\left(S_{i\;+\;1}=\text{P}|S_{i}=\text{P}\right)\;=\left(\frac{m}{M-1}\right)\left(\frac{M}{N}\right) \end{array}$$

where *M* is the number of times a predictive response happened, *m* is a number of times a predictive response was followed by another predictive saccade and *N* is the total number of saccades. In this case the predictive response is considered every saccades with latency in a range of −300–80 ms in regard to the stimulus on-set. The conditional probability was calculated for each subject and than the *t*-test was used to analyze the between group difference.

## Results

In the cognitive assessment, the results showed no difference between groups for the IQ and attention scores, which was expected because both tests were used as exclusion criteria. On the reading/writing tests, groups were compared based on total score and time needed to complete the task. It is important to point out that usually only a test score but not the execution time is reported in studies with dyslexia. We opt to report both in order to show the interaction between the speed and error rate in different tasks. Statistical comparisons showed that DG needed significantly more time to complete the word reading task but showed a tendency for an error rate in the same test. DG had also statistically significant lower score for word writing and showed tendency for the orthography checking score. The execution speed was not significant in either of the tests. In the comprehension task, no difference in score or time was found when subjects were listening to the sentences, but when the sentences were read, DG took significantly longer time, although the score was on the same level as CG. This is not surprising since the Sentence reading comprehension is based on silent sentence reading and a response selection from out of five supporting pictures. Rapid naming task also showed longer time for DG. No difference was found in the Phonological awareness test, but further analyses showed that there was a ceiling effect because in most of the subtests covering syllable addition and subtraction, phoneme addition and subtraction, etc., the majority of the children achieved a full score. The results are described in Table 1.

**Table 1:** **Results (the mean and standard deviation) of the psychometric tests of the dyslexic and control subjects tested in this study**.

**Cognitive Assessment _[DF]_**	**Measure**	**Dyslexic children Mean (SD)**	**Controls Mean (SD)**	***F***	***p***	***d***
General intelligence (WISC)_[1, 28]_	Total IQ	116 (3)	112 (3)	0.863	*0.361*	*0.146*
	Verbal IQ	113 (4)	110 (4)	0.587	*0.450*	*0.115*
	Executive QI	116 (4)	114 (4)	0.143	*0.708*	*0.065*
Continuous attention_[1, 26]_	Score	56 (14)	59 (15)	0.184	*0.672*	*0.070*
Word writing_[1, 26]_	Time (seg) Score	1573 (920)	1259 (640)	1.096	*0.305*	*0.172*
		24 (4)	29 (5)	6.560	***0.017***	***0.693***
Word orthography checking_[1, 26]_	Time (seg) Score	403 (132)	372 (402)	0.077	*0.784*	*0.058*
		60 (5)	64 (6)	3.603	***0.069***	***0.448***
Word reading_[1, 26]_	Time (seg) Score	429 (139)	332 (75)	5.219	***0.031***	***0.595***
		33 (3)	35 (1)	3.418	***0.076***	***0.429***
Sentence listening comprehension[1, 26]	Time (seg) Score	651 (133)	618 (189)	0.271	*0.607*	*0.079*
		39 (2)	38 (2)	0.658	*0.425*	*0.122*
Sentence reading comprehension_[1, 26]_	Time (seg) Score	1959 (1048)	1104 (183)	9.037	***0.006***	***0.825***
		37 (4)	37 (5)	0.072	*0.790*	*0.058*
Phonological awareness_[1, 27]_	Time (seg) Score	2432 (490)	2140 (504)	2.449	*0.126*	*0.332*
		38 (7)	38 (7)	0.010	*0.921*	*0.051*
Rapid naming_[1, 24]_	Time (seg) Errors	38 (12)	30 (5)	4.033	***0.056***	***0.487***
		0.36 (0.63)	0.58 (1.24)	0.359	*0.555*	*0.089*

*Statistically significant p-values are highlighted in bold*.

In the **Visually guided saccades** task, the groups comparison showed no difference for saccadic latencies [*F*_(1, 24)__=_0.01; *p* = 0.92; DG_Lat_ = 256 ms, *SD* = 32 ms; CG_Lat_ = 255 ms, *SD* = 35 ms]. The ANOVA 2 × 2 test with group and side (left/right) as the between-subjects factor showed a tendency for side, with both groups producing rightward saccades in shorter latencies [*F*_(1, 24)__=_3.625; *p* = 0.06; DG_*Left*_ = 271 ms, *SD* = 43 ms; DG_*Right*_ = 247 ms, *SD* = 33 ms; CG_*Left*_ = 263 ms, *SD* = 38 ms; CG_*Right*_ = 246 ms, *SD* = 42 ms]. No difference was found for the group factor or group × side interaction.

**Figure 2:**
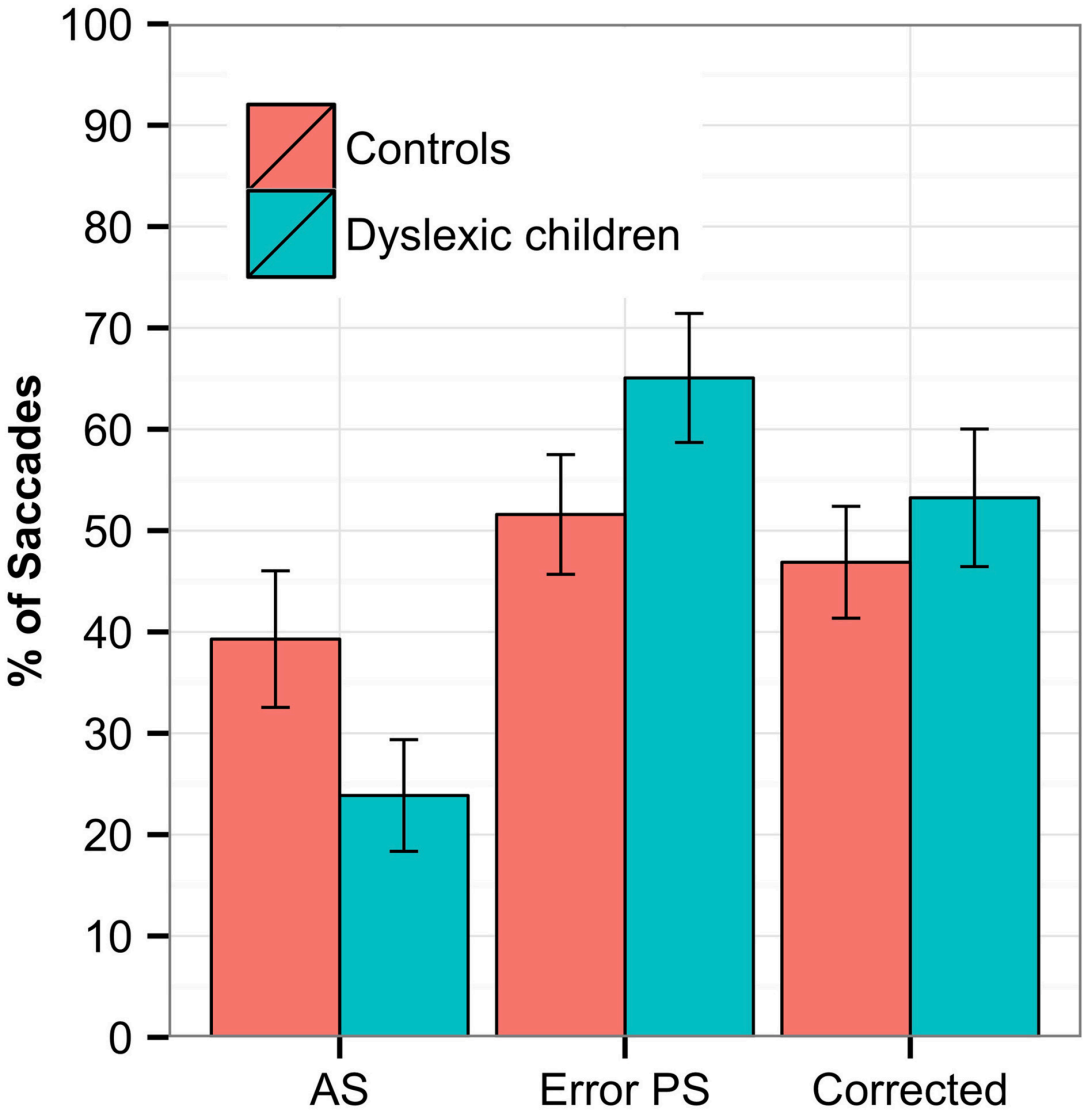
**The Antisaccades task with the percentage of correct antisaccades (AS), error pro-saccades (PS, saccades in the direction of the stimuli) and corrected error (pro-saccade followed by correct antisaccade)**. Statistically significant differences were found between groups for correct antisaccades (*p* < 0.000) and error pro-saccades (*p* < 0.05). The error bars represent standard error.

In the **Antisaccades** task, a regression was conducted on the proportion of correct antisaccades, error pro-saccades and corrected antisaccades. Both groups showed a very low proportion of correct antisaccades, with DG scoring significantly less than CG (estimate = 0.064, *SE* = 0.016, *z* = 3.874, *p* < 0.001 *DG* = 25%, *CC* = 37%). A small proportion of antisaccades indicates that both groups committed errors in the task—generating a pro-saccade after the stimuli onset instead of the required antisaccades; this occurred in DG more than CG (estimate = −0.0428, *SE* = 0.015, *z* = −2.802, *p* = 0.005, *DG* = 63%, *CC* = 54%). In spite of making more errors, no between-group difference was found for DG in the rate of corrections compared to CG (estimate = −0.004, *SE* = 0.015, *z* = −0.323, *p* = 0.747, *DG* = 50%, *CC* = 49%). We checked whether the correct antisaccades or errors were more frequent for either sides (left/right) or between groups, but no group × side interaction was found. No between-group difference was found for the latency in correct antisaccades (*DG* = 344 ms *SD* = 87 ms, *CC* = 345 ms, *SD* = 60 ms), the error pro-saccades (*DG* = 212 ms *SD* = 27 ms, *CC* = 213 ms, *SD* = 29 ms), or the corrected antisaccades (*DG* = 585 ms *SD* = 81 ms, *CC* = 595 ms, *SD* = 116 ms). The rates of antisaccades, error pro-saccades and corrected antisaccades are depicted in Figure 2.

In the **Predictive saccades** task, a regression was performed on the proportion of saccades within each type, which yielded statistically significant differences for predictive (estimate = 0.059, SE = 0.016, *z* = 3.747, *p* < 0.001, *DG* = 34%, *CG* = 46%) and regular saccades (estimate = −0.043, SE = 0.016, *z* = −2.794, *p* < 0.01, *DG* = 47%, *CG* = 37%).

**Figure 3:**
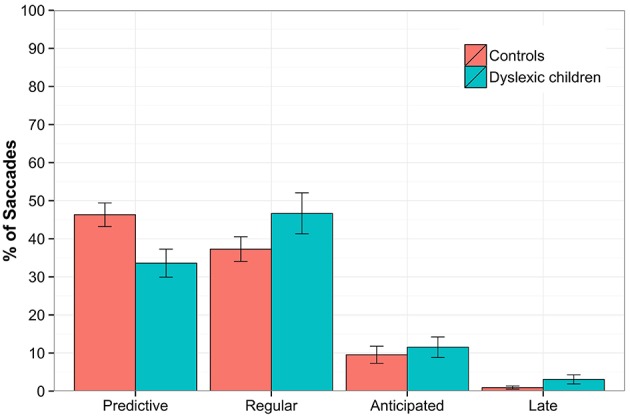
**The Predictive saccades task**. The percentage of saccades plotted for the groups according to the latency as predictive (−300–120 ms), regular (120–300 ms), anticipated (< −300 ms) and late (>300 ms). Statistically significant differences were found between groups for predictive (*p* < 0.001) and regular saccades (*p* < 0.05). The error bars represent standard error.

No differences were found for error saccades, but DG performed more late saccades than CG (estimate = −0.135, *SE* = 0.064, *z* = −2.117, *p* < 0.05, *DG* = 3%, *CG* = 0.9%). The results are depicted in Figure 3.

Comparing the latency of predictive and regular saccades between groups, a statistical tendency was found for predictive saccades [*F*_(1, 28)_ = 3.08; *p* = 0.09, DG_Lat_ = −70 ms, *SD* = 60 ms; CG_Lat_ = −103 ms, *SD* = 42 ms], with DG showing shorter saccadic latencies than CG, but no group effect was found for regular saccades.

**Figure 4:**
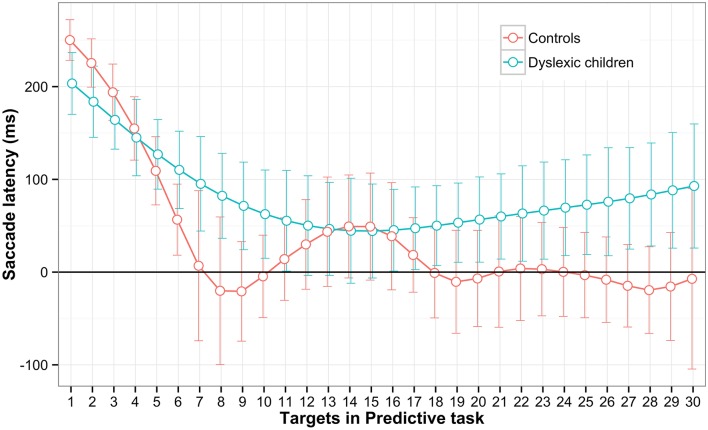
**Saccadic latencies for each target in the Predictive task**. The mean saccadic latencies within predictive and regular type were averaged for every target along the block. The data were smooth with the Cubic smooth splines and the confidence interval was estimated using 10,000 bootstrap replicates (error bar).

To see the learning effect of predictive saccades along the task block, the subjects' means were averaged and interpolated for each stimulus and are plotted in Figure 4. The data showed that both groups started predicting after the third stimulus and continued to reduce the latency as the task progressed. However only the CGs' learning indicated prediction efficiency toward the end of the block with the latency approaching zero time on x-axis and remaining within the predictive time range −300–80 ms. To support this argument statistically, the groups were compared for the probability estimate of remaining within the predictive state. There was a significant difference between the DG (*M* = 0.192, *SD* = 0.165) and CG (*M* = 0.329, *SD* = 0.135) groups; *t*_(28)_ = −2.48, *p* = 0.02. This indicate that CG is more efficient in making a sequence of saccades with predictive latencies.

## Discussion

In this study, the oculomotor behavior of children with developmental dyslexia was similar to that of the controls in visually guided saccades, but showed altered patterns in antisaccades and predictive saccades. In the antisaccades task, the dyslexic children showed lower rate of correct antisaccades. In the predictive saccades task, dyslexic children did not achieve predictive efficiency on the level of the controls, as they made more regular than predictive saccades, and the learning curve represented by reduction in saccadic latency as the task progressed was less evident. In the visually guided saccades task, no group difference was found, but both groups showed shorter latencies to the target appearing on the right side. The results are discussed below.

Over the last decades, a few studies have investigated oculomotor behavior in children with developmental dyslexia and normal reading controls. Our results are coherent with those showing dyslexics' impairment in cognitively more complex tasks, such as antisaccades and predictive saccades, but not basic oculomotor behavior, such as visually guided saccades (Olson et al., 1991). Impaired performance was found in a study of Biscaldi et al. (2000) that assessed 506 dyslexics and 114 controls, divided into four groups from 7 to 17 years of age. Children with dyslexia showed a lower number of correct antisaccades and corrections and a higher number of misses (lost trials), with the increasing difference among the age groups (Biscaldi et al., 2000). Our results agree with these data, although a somewhat lower rate of correct antisaccades was found in children with dyslexia for the corresponding age groups (8–13 years old). Our children scored on the level corresponding to the group of 8-year-olds. Similar to the literature, no group difference was found in the latency of correct antisaccades (Fischer and Weber, 1997; Biscaldi et al., 2000; Seassau and Bucci, 2013). In Biscaldi et al.'s (2000) study, the antisaccades task was presented with a gap, a small interval of time between the central point offset and the lateral target onset. Due to this important methodological difference between the two studies, one would expect a lower rate of errors and longer latencies in the overlap task, but in fact, a higher rate of error and similar latency was found in our overlap design (Biscaldi et al., 2000). Because the gap/overlap effect is still controversial in children, the performance variability in this age/condition may be responsible for this difference (Eenshuistra et al., 2007). On the other hand, the findings in the antisaccades task are in agreement with the reports on healthy subjects' performance in the same task. Luna and Sweeney (2001) found that the antisaccades task had a high rate of error, approximately 50–60% of trials, in children and became more stable approximately 15 years of age. The rates of our control group are within this range, although the dyslexic group is over the edge.

The novel finding of this study refers to the reduced number of predictive saccades and predictive learning in dyslexic children. Predictive saccades offer a good design for procedural motor learning and can provide some knowledge of adaptation abilities in clinical populations (Smit and Van Gisbergen, 1989). To the best of our knowledge, only one study assessed predictive tracking in normally developing children for varying frequencies of the target movement. In this study, children showed longer latencies than adults, but only in the mid-range frequencies, such as 0.75, 1.00, and 1.25 Hz. The 1 Hz stimuli frequency yielded a latency distribution with a peek approximately 100 ms in healthy children, which is comparable to our results. Ross and Ross (1987) found longer latencies for healthy children than for adults and argued that the slowing of oculomotor response could be attributed to immaturity, while cognitive processing behind the task operation was similar. Considering our results of healthy and impaired children, dyslexics made less predictive saccades, but did not differ in regard to regular saccades latency. Thus, we argue that procedural learning in dyslexic children is altered and differ from a developmental curve found in normally developing children. Our results are coherent with procedural learning outcome found in dyslexic children performing the serial reaction time task (SRT). A meta-analysis of 14 studies with SRT showed worse procedural learning abilities in subjects with dyslexia and the participant's age was associated to the heterogeneity of the results, which means that the difference in learning efficiency was mainly present in children and to smaller degree in adults with and without dyslexia (Lum et al., 2013).

According to a model of predictive tracking, the reduction in latency is possible due to the internal stimulus timing representation (internal clock), the memory of the previous saccade intervals and error analyses of the past and future eye movements (Joiner and Shelhamer, 2006). Our data do not allow further insight into a possible impairment of the model components in dyslexic children, but different shapes of the learning curve indicate difference in the way both groups (CG and DG) deal with predictive saccades. It has been argued that internal timing skills may be altered in dyslexics for a variety of motor and/or musical tasks (Birkett and Talcott, 2012; Goswami, 2014), and we suggest that implicit learning of visual stimulus timing may also be impaired in the predictive tracking. Also visual memory span impairment has been reported in previous studies for dyslexics and would further lower predictive tracking efficiency (Vicari, 2005; Stoodley et al., 2006; Menghini et al., 2011). Based on the curve shape in the control children, we suggest that visual predictive learning is not a linear process but rather step-like construction of timing and spatial internal representation passed onto the central executive system. Further examination of neural correlates by functional imaging techniques can provide more evidence on whether the behavioral differences found in predictive saccades stem from different neuro-functional systems.

In spite of the need for further research on this subject, the findings of this study could have important implications for intervention in children diagnosed with dyslexia. First, the error-detection mechanism, as an implicit and fast operating process that identifies similarities in the past and future events, could benefit from training of predictive saccades. Some studies showed a decrease in error-rate followed training on anti-saccade tasks, and the effect type was congruent with the kind of an exercise performed in healthy subjects (Dyckman and McDowell, 2005) and children with dyslexia (Fischer and Hartnegg, 2000). Second, the predictive saccades task offers a possibility of improving the timing representation in visual domain, that could be also transposed to the auditory domain, that has been tested in rhythm training in studies with musical interventions (Habib et al., 2016). The question that remains to be answered is to what amount these improvements can transfer to the reading and/or spelling skills and exactly who can benefit form them. Fischer and Hartnegg (2000) showed that only about a third out of 86 children with dyslexia improved reading scores and hand writing after oculomotor training with antissacades task.

To conclude, our results indicate similar behavior between dyslexic and control children in basic oculomotor tasks, such as visually guided saccades, but impairment in oculomotor properties related to more complex cognition. This may involve deficient implicit learning of time/position patterns and/or error analyses of the past and future eye movements. The concern of this paper was not to answer whether this deficit is a cause or a consequence of developmental dyslexia, but rather to confirm and extend inconsistent findings on oculomotor patterns. This was achieved by showing impaired internally guided saccades (predictive saccades) and volitional saccades with inhibition (antisaccades).

## Author contributions

All authors listed, have made substantial, direct and intellectual contribution to the work, and approved it for publication.

## Funding

This work was supported by grant for the last author EM from CNPq (No. 309453/2011-9).

### Conflict of interest statement

The authors declare that the research was conducted in the absence of any commercial or financial relationships that could be construed as a potential conflict of interest.
